# Successful Management of Acute Promyelocytic Leukemia in a Patient Who Presented With Acute Ischemic Stroke on Top of Subdural Hematoma

**DOI:** 10.7759/cureus.45243

**Published:** 2023-09-14

**Authors:** Shatha Mallah, Fahed Owda, Hamza Hamayel, Ahmad Enaya, Osama Mallah, Dina Abugaber, Razan Odeh

**Affiliations:** 1 Department of Internal Medicine, Faculty of Medicine and Health Sciences, An-Najah National University, Nablus, PSE; 2 Department of Internal Medicine, An-Najah National University Hospital, Nablus, PSE; 3 Department of Radiology, An-Najah National University Hospital, Nablus, PSE; 4 Department of Medicine, Faculty of Medicine and Health Sciences, An-Najah National University, Nablus, PSE; 5 Department of Hemato-oncology, An-Najah National University Hospital, Nablus, PSE

**Keywords:** disseminated intravascular coagulation (dic), all-trans retinoic acid (atra), case report, ischemic stroke, subdural hematoma, aml-m3, apl, aml, acute promyelocytic leukemia

## Abstract

Acute promyelocytic leukemia (APL), a distinct subtype of acute myelogenous leukemia (AML), is commonly associated with a heightened risk of bleeding due to coagulopathy. Thrombotic events, although less frequent, have also been linked to APL. However, the occurrence of ischemic stroke as an initial presentation of APL, particularly concomitant with central nervous system (CNS) bleeding, is exceedingly rare. The combination of these two complications is not reported in APL patients and is anticipated to carry a high mortality rate even with treatment. In this report, we describe the case of a young female patient with no significant medical history, who presented with decreased consciousness and recurrent seizures. Brain magnetic resonance imaging (MRI) revealed the simultaneous occurrence of acute ischemic stroke and acute-on-chronic subdural hematoma. The subsequent bone marrow biopsy confirmed the diagnosis of APL, displaying the characteristic positive promyelocytic leukemia (PML)-retinoic acid receptor alpha (RARA) t(15;17) translocation. The patient was promptly initiated on a high-risk AML-M3 protocol, coupled with supportive treatment through platelet transfusion. Remarkably, a favorable response to treatment was observed, and a marked improvement in her neurological parameters was observed within 2 weeks duration of treatment. Subsequent assessment through a bone marrow biopsy one month later revealed complete remission, with the PML-RARA fusion gene becoming negative following a single course of consolidation therapy.

## Introduction

Acute promyelocytic leukemia (APL) is an aggressive hematologic malignancy and a distinct variant of acute myelogenous leukemia (AML), historically classified as AML-M3 according to the French-American-British classification [1]. The characteristic cytogenetic abnormality of APL involves a reciprocal translocation between chromosomes 15 and 17, known as t(15;17), which is observed in over 95% of APL cases. This translocation results in a fusion product between two specific genes: the promyelocytic leukemia (PML) gene from chromosome 15 and the retinoic acid receptor alpha (RARA) gene from chromosome 17 [2].

APL poses a significant medical emergency, as early mortality rates are high in the absence of appropriate treatment. Without timely administration of all-trans retinoic acid (ATRA), the median survival of patients is less than one month [3]. Although survival outcomes have improved with treatment, early death remains a significant concern, often attributed to hemorrhage, differentiation syndrome, and infection [4].

Thrombotic complications in acute leukemia are relatively rare and often overshadowed by bleeding and infection-related issues that dominate the clinical presentation [5]. Thrombotic events are typically observed during or after the initiation of ATRA treatment and are infrequently seen as the initial manifestation of the disease [6]. In a retrospective review of 63 APL patients, only one patient (1.6%) was reported to have experienced thrombosis before receiving chemotherapy with ATRA [7]. Thrombosis can affect any organ and involve either the arterial or venous systems. Ischemic stroke, as an exceptionally rare initial presentation of APL, is associated with a significantly high mortality rate [8].

Early treatment with ATRA serves as the cornerstone of APL management, with the addition of arsenic trioxide and/or chemotherapy playing a crucial role in achieving complete remission [9]. To date, no cases have been reported in the literature of APL with concomitant central nervous system (CNS) bleeding and thrombosis. In this report, we present an exceedingly rare case of a female in her late twenties who presented with acute ischemic stroke on top of subdural hemorrhage as the initial clinical manifestation of APL.

## Case presentation

A previously healthy 28-year-old female was referred to our hospital for further hematologic investigation and treatment following the discovery of a subdural hematoma and abnormal cell counts. Her symptoms initially manifested two months before admission with the sudden onset of widespread bruising within one week. At that time, a complete blood count (CBC) revealed hemoglobin (Hb) of 7.4 g/dL, white blood cells (WBCs) of 17 K/μL, and platelets of 14×10^9/L. Despite the abnormal findings, she did not exhibit signs of sepsis or significant bleeding during that period. However, one week prior to transfer, she developed severe headaches and vomiting, prompting a brain computed tomography (CT) scan that confirmed the presence of a subdural hemorrhage. The patient remained conscious and was managed conservatively through platelet transfusions.

Upon arrival at our hospital, the patient presented as conscious, oriented, and hemodynamically stable. She denied experiencing headaches or other neurological symptoms. Physical examination revealed multiple bruises scattered across her body, while laboratory investigation indicated leukocytosis with anemia and thrombocytopenia (Hb=8.4 g/dL, WBCs=15 K/μL, and platelets=30×10^9/L). Coagulation profile results displayed an international normalized ratio (INR) of 1.1, activated partial thromboplastin time (aPTT) of 45 seconds, a D-dimer level of 7 mg/L, and a fibrinogen level of 120 mg/dL. Blood films were obtained, and a bone marrow biopsy with aspiration was performed.

**Figure 1:**
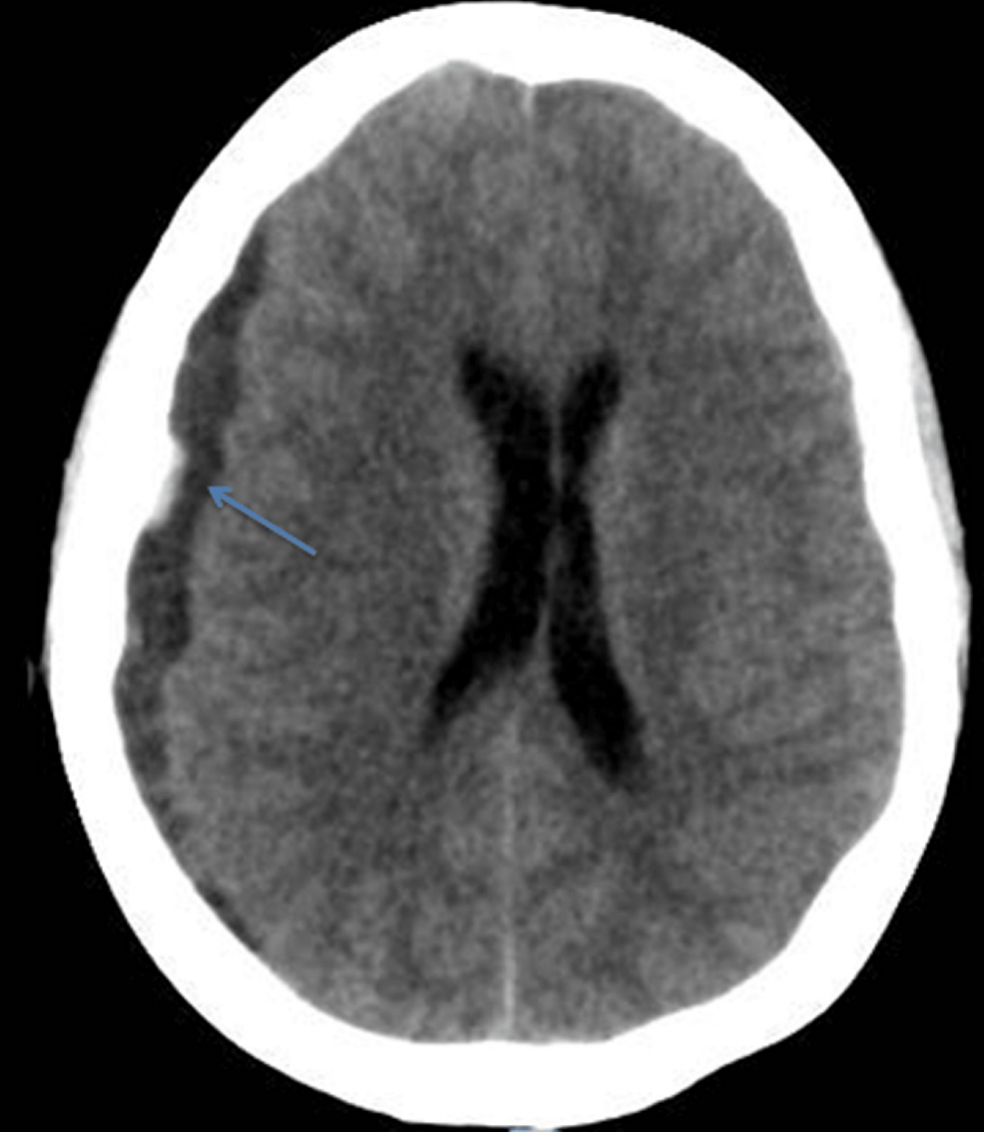
Acute on top of chronic subdural hematoma. Non-contrast CT scan (transverse view) showing chronic (hypodense) right-sided subdural hematoma (blue arrow) with a minimal hyperdensity.

Supportive management was initiated, aiming for a target platelet count above 30,000. However, a few hours after admission, the patient experienced a severe headache followed by a decrease in consciousness (Glasgow Coma Scale=8), and anisocoria was noted upon physical examination. To protect her airway, she required intubation. A brain CT scan with and without intravenous (IV) contrast revealed a chronic right-sided subdural hematoma measuring approximately 1 cm in maximal thickness, along with a 6 mm midline shift to the left and effacement of cerebral cortical sulci. Additionally, the scan indicated a minimal hyperintense area suggestive of an acute event on top of the chronic hematoma (Figure 1).

**Figure 2:**
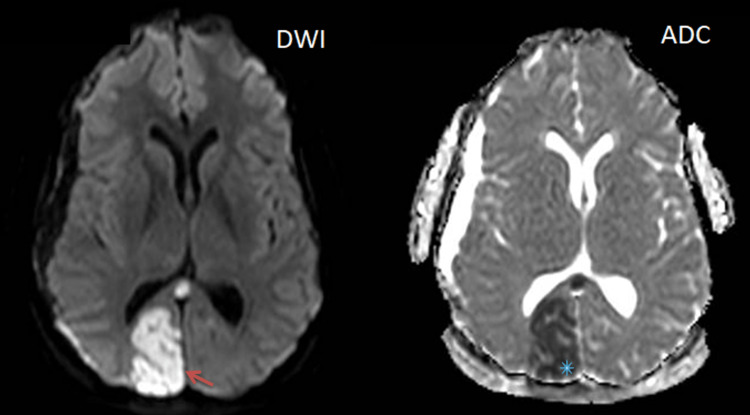
Acute ischemic stroke of right PCA territory, in addition to chronic right subdural hematoma. MRI (transverse view) showing the area of restricted diffusion in the medial aspect of the right occipital lobe that appears hyperintense on DWI (red arrow) and hypointense on ADC mapping (asterisk), with a right-sided subdural collection that follows CSF. PCA: posterior cerebral artery, MRI: magnetic resonance imaging, DWI: diffusion-weighted imaging, ADC: apparent diffusion coefficient, CSF: cerebrospinal fluid.

On repeated physical examination, her pupils were reactive to light and bilaterally symmetrical in size, but she remained unconscious. As the CT scan couldn't fully explain her new clinical picture, she underwent brain magnetic resonance imaging (MRI) with IV contrast, revealing acute infarction in the territory of the right posterior cerebral artery (PCA) in addition to the right subdural hematoma. The MRI also identified a minimal late subacute subdural hematoma in the left temporal region (Figure 2).

Investigations into potential underlying causes of the ischemic stroke, including an electrocardiogram (ECG), transesophageal echocardiogram (TEE), and CT angiogram, yielded unremarkable results. Thrombophilia and vasculitis workups were within normal limits. Consequently, the patient's ischemic stroke was attributed to disseminated intravascular coagulation (DIC) induced by APL.

**Figure 3:**
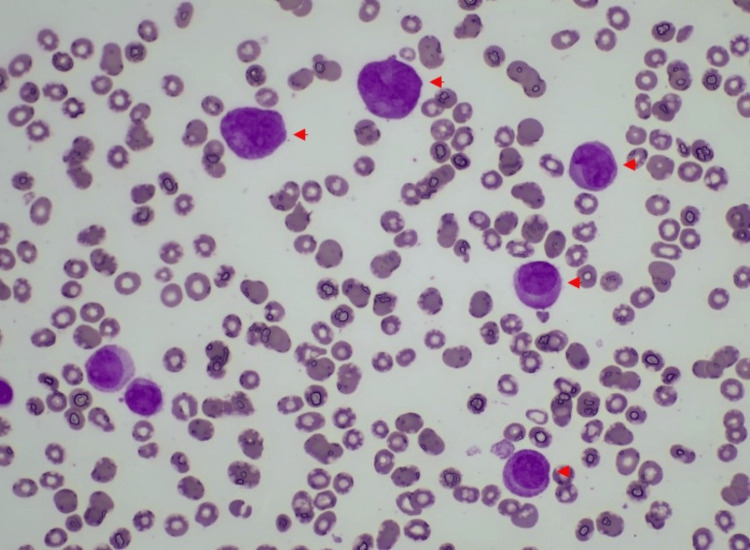
Bone marrow aspirate predominated by abnormal promyelocytes (highlighted with red arrowheads) stained with Giemsa at 600x magnification.

**Figure 4:**
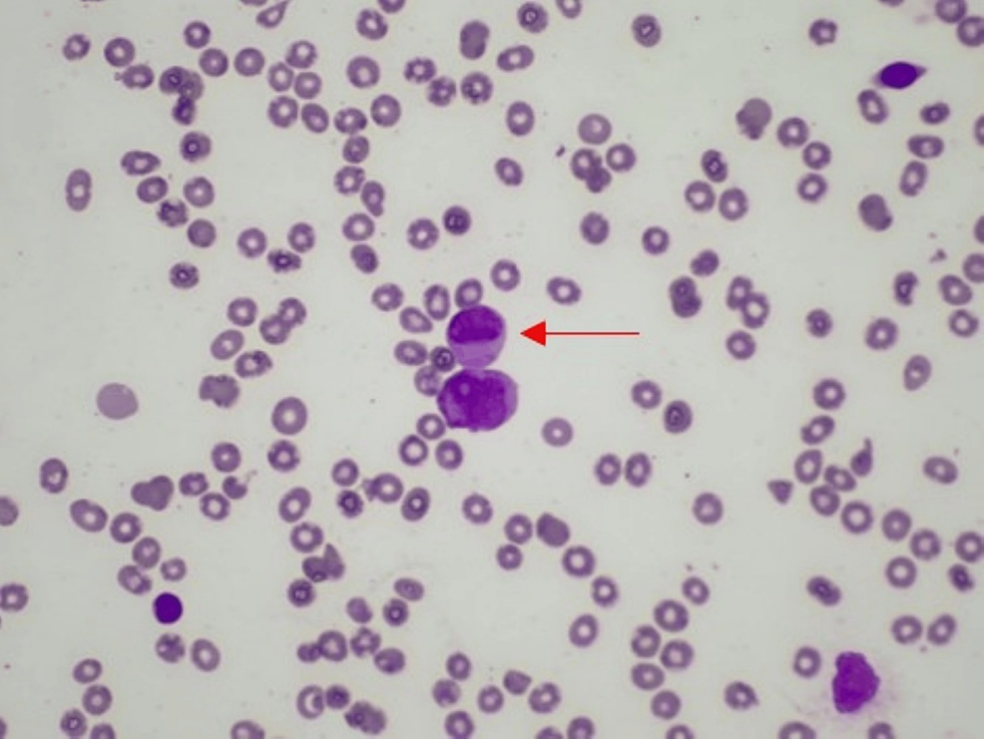
Bone marrow aspirate showing an occasional promyelocyte with intracytoplasmic Auer rods (red arrow).

A multidisciplinary team, consisting of a hemato-oncologist, neurologist, and neurosurgeon, engaged in a comprehensive discussion regarding the optimal course of action. It was decided to pursue conservative management, which included regular platelet and cryoprecipitate transfusions with platelets target above 50,000, and efforts to reduce intracranial pressure (ICP) by maintaining sodium levels within upper limits and carbon dioxide (CO2) levels within lower limits. Simultaneously, the patient was initiated on a high-risk APL protocol with ATRA 45 mg/m2 administered daily in two divided doses, along with daunorubicin and dexamethasone 10 mg IV daily to prevent differentiation syndrome. Continuous hourly neurological assessments were performed, and intervention was planned in the event of neurological deterioration. The following day, the results of the bone marrow biopsy showed abnormal neoplastic promyelocytes with occasional Auer rods (Figures 3, 4) and reverse transcriptase-polymerase chain reaction (RT-PCR) testing for t(15;17)(q22;q12) of the PML-RARA fusion gene was positive confirming the diagnosis of APL.

Throughout the initial days of the patient's intensive care unit (ICU) stay, she remained connected to a mechanical ventilator in minimal settings, and her blood pressure was controlled using labetalol infusion to maintain systolic blood pressure (SBP) between 140-160 mmHg. She received light sedation through fentanyl infusion. Despite regular platelet transfusions (approximately 15 units daily), her platelet counts ranged from 20,000 to 70,000. Attempts to wean her off sedation were unsuccessful due to agitation, and an electroencephalogram (EEG) showed no seizure activity. Hence, she continued under ventilator support with light sedation.

After a few days, the patient's level of consciousness began to gradually improve, although she was not yet eligible for extubation. Her course was complicated by a further drop in platelet counts, a known side effect of treatment. The lowest platelet count, reaching 8X10^9/L, occurred within 7 to 14 days of receiving daunorubicin, despite regular platelet transfusions. Fortunately, her neurological status remained stable, and no significant bleeding episodes were observed.

**Figure 5:**
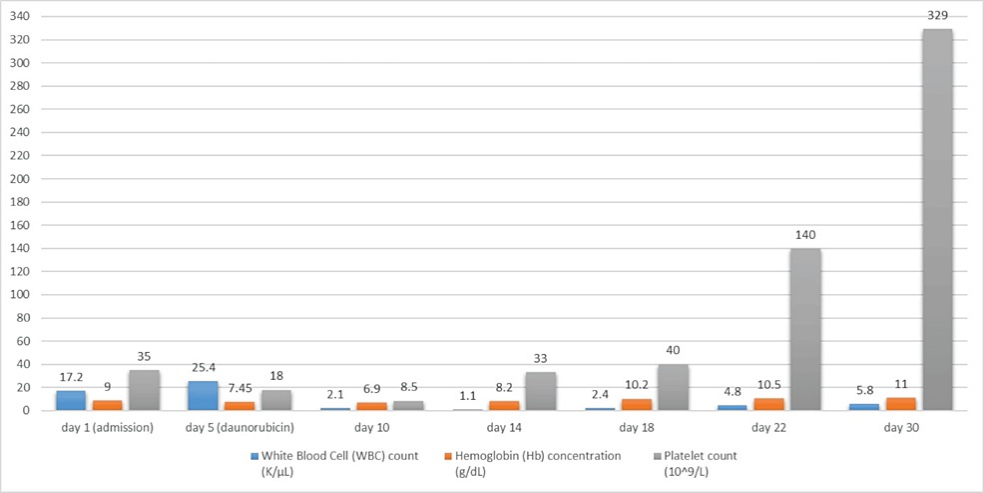
The timeline of the patient's white blood cell count, hemoglobin concentration, and platelet count from initial presentation through the course of her admission until complete recovery.

During the second week of the patient's ICU admission, she developed neutropenic fever and her course was complicated by ventilator-associated pneumonia. She was given empirical antibiotics of vancomycin and piperacillin-tazobactam. Vancomycin was discontinued after 3 days according to culture and sensitivity results, and she was effectively managed with piperacillin-tazobactam. As the second week progressed, she experienced a gradual improvement in consciousness and a concurrent recovery of her blood counts. Subsequent imaging in the form of a brain CT scan revealed a reduction in the subdural hematoma size to 7 mm, accompanied by the resolution of midline shift. Furthermore, the DIC workup conducted in the second week yielded negative results. By the third week of her hospitalization, the patient's blood counts had fully recovered (Figure 5). Throughout her treatment, she received approximately 300 units of platelets, 19 units of packed red blood cells, and four units of fresh frozen plasma. A repeated bone marrow biopsy performed one month after admission confirmed the achievement of complete morphological and molecular remission.

## Discussion

The clinical features of AML are typically attributed to bone marrow failure or leukemic infiltration of other organs, resulting in signs and symptoms associated with pancytopenia, such as pallor, weakness, bleeding tendency, skin lesions, fever, and frequent infections. APL, a distinct subtype of AML, presents with unique complications, with DIC being a more frequent occurrence in this subtype. DIC in APL is often associated with a high risk of bleeding and, to a lesser degree, thrombotic events [10]. Our case report observed an exceptional initial presentation of APL as CNS bleeding and thrombosis, manifested by subdural hematoma and ischemic stroke, respectively.

Although arterial and venous thrombosis as presenting features of APL are uncommon, they have been reported in previous studies. Thrombosis was reported in approximately 20% of APL patients in one observational study, with only 1.3% of cases occurring prior to the initiation of chemotherapy [7]. Another study reported that around 9.6% of APL patients presented with thrombotic events [11]. Several studies have highlighted the risk of thrombosis during induction chemotherapy with ATRA, as its administration has been associated with life-threatening venous thromboembolic events in APL patients. It has been observed that the initiation of ATRA treatment can lead to a prothrombotic state by disrupting the balance between the procoagulant and fibrinolytic properties of leukemic promyelocytes, thus resulting in clinically significant thrombotic events [12]. In our case, the occurrence of ischemic stroke, although rare compared to other thrombotic events, carries a high mortality rate as reported in the literature [13]. The simultaneous presence of CNS thrombosis and bleeding in APL patients may be considered an ominous and challenging presentation, although data on mortality specifically for this scenario is limited.

The thrombogenicity of APL is believed to result from a combination of complex factors, including the activation of the extrinsic pathway of coagulation due to the release of procoagulant substances from neoplastic cells such as tissue factor, cancer procoagulant, and membrane factor V receptor, which play a crucial role in DIC development [14]. Additionally, the increased expression of annexin II receptor on the surface of leukemic promyelocytes leads to enhanced binding of tissue plasminogen activator and increased plasmin generation, ultimately contributing to enhanced fibrinolysis and an elevated bleeding tendency in APL patients [15].

Most APL cases arise from a balanced reciprocal translocation between chromosomes 15 and 17, resulting in the formation of the PML-RARA fusion gene [2]. This fusion gene has become a pivotal therapeutic target for ATRA, which has revolutionized the treatment of APL. ATRA induces apoptosis of malignant APL cells and promotes the differentiation process of promyelocytes into mature granulocytes [16]. Initiation of ATRA therapy in patients with suspected APL, without waiting for genetic confirmation, along with aggressive blood product support during induction, is highly recommended [17]. ATRA treatment increases the levels of thrombomodulin, reduces the production of tissue factor and cancer procoagulant by leukemic cells, and down-regulates the expression of annexin II cell surface receptors in APL malignant cells, resulting in a notable improvement in the coagulopathy associated with this malignant disorder [18].

In our patient's case, after ruling out other potential causes of ischemic stroke, including cardioembolic stroke, vasculitis, and hypercoagulable states, the presentation was attributed to DIC as a complication of APL. Thus, APL management became the cornerstone of DIC treatment. Despite data indicating that starting ATRA may trigger thrombosis, it remained our patient's most reliable management option and was not discontinued upon the diagnosis of ischemic stroke. Additionally, administering daunorubicin to achieve complete remission, despite its adverse effect on platelet counts, was deemed essential in this patient's care due to the high WBC counts observed. To minimize the risk of bleeding, the patient received regular platelet and cryoprecipitate transfusions.

Current guidelines recommend surgical evacuation of chronic subdural hematomas measuring more than 10 mm in thickness or cases with a midline shift exceeding 5 mm [19]. Although our patient presented with a subdural hematoma and a 6 mm midline shift, multiple factors favored conservative management over surgical intervention. Firstly, the patient had exceptionally low platelet counts, making surgery a significant bleeding risk. Furthermore, CT imaging performed when the patient had lost consciousness revealed a minimal acute subdural hematoma (< 1 mm) on top of a chronic stable subdural hematoma, which did not fully explain her neurological deterioration. Lastly, an MRI showed a new ischemic stroke in the right PCA territory, suggesting that the clinical deterioration and increased intracranial pressure resulted from CNS thrombosis. Considering these factors, surgical intervention for the subdural hematoma was considered a less favorable option due to the potential risks outweighing the desired benefits.

## Conclusions

In conclusion, this unique case sheds light on the rare occurrence of concurrent central nervous system (CNS) bleeding and thrombosis in a patient with acute promyelocytic leukemia (APL). Healthcare practitioners should be cognizant of this challenging clinical dilemma and manage it cautiously, considering all associated risks. Our case demonstrates that conservative management and early administration of all-trans retinoic acid (ATRA) yielded successful outcomes. Vigilance, multidisciplinary collaboration, and further research are necessary to enhance our understanding and optimize the management of this complex clinical entity.
